# A Call for More Rigor in Science and Health Communication

**DOI:** 10.3390/ijerph19031825

**Published:** 2022-02-05

**Authors:** Rima E. Rudd

**Affiliations:** Department of Social and Behavioral Sciences, Harvard T.H. Chan School of Public Health, 677 Huntington Avenue, Boston, MA 02115, USA; rrudd@hsph.harvard.edu

**Keywords:** environmental health literacy, health literacy, formative research

## Abstract

Successful dissemination of scientific knowledge relies on the ability of the writer, speaker, and designer to provide information and data that is both available and accessible to the audience for whom it is intended. Scientific rigor, uniformly applied to the development of medicines, products, and devices must be applied, as well, to communications—spoken, written, posted, or displayed. Rigorous development and design protocols call for formative research data gathering, careful pilot testing with members of the intended audience, needed revisions, and rigorous assessments. Guidelines and tools developed for health literacy applications can be adopted and adapted for environmental health research and educational efforts in the design of questionnaires, instructions, education and report back materials, as well as for public discourse.

## 1. Introduction

Public skepticism related to scientific findings about issues such as climate change, disaster mitigation and management, the health-related effects of the natural and built environments, or the COVID pandemic has become a major challenge. More than five years ago, two reports from the National Academies called upon the scientific community to help create a social environment in which scientific expertise is trusted and science literacy can thrive. Both reports, the 2016 Science Literacy: Concepts, Contexts, and Consequences [1] and the 2017 Communicating Science effectively: A Research Agenda [2] call upon scientists to do a better job communicating scientific complexity and nuance so that findings are understood by and useful for the intended audience. Both reports acknowledge that information alone does not drive efficacious change, but that information is valued as a prerequisite. Openness and sharing, the reports remind us, build trust between the members of the public and those who generate and disseminate knowledge—scientists, researchers, policy makers, practitioners, and publishers.

The concept of democratization of knowledge emphasizes the importance of the information exchange and of wide spread dissemination to create and build knowledge. However, there is an important distinction to be made between information that is readily *available* and information that is *accessible.* Readily available information—presented in texts and booklets, posted online, spoken over the air waves, reported in journals, magazines, and newspapers—is not necessarily accessible and usable. Unfamiliar vocabulary, uncommon measures, dense texts, and confusing graphs or charts can render available information inaccessible. Critical health information that is not clear to the lay public cannot build knowledge, but will, instead, inhibit use of provided information and subsequent action.

Increasingly, government plans and policies have been calling for, and in some instances requiring, clearly written and more accessible information. For example, in the U.S the Plain Language Act of the Congress requires that government agency issued materials for the public use plain language [3]. The 2010 Health Literacy Action Plan from the U.S. Department of Health and Human Services (USDHHS) calls for governmental and institutional responsibility and action for making health and safety information accessible [4]. The recent conceptualization of health literacy goals and objectives for the U.S. document Healthy People 2030 calls for organizations and professionals to eliminate literacy related barriers to information and care [5]. Similarly, governmental initiatives in Canada, many countries of Europe, as well as in Australia and New Zealand have been calling for in, and in some cases requiring, clearly written and more easily accessible information [6].

Environmental health communication, as is true for most science communication, can be challenging. Educational materials, public reports, and calls to action rely on sophisticated literacy and numeracy skills of the audience. Concepts, technical terms, or measures are often not presented in everyday words, with explanations, or in a familiar format. This discussion is an attempt to make an argument for needed rigor in communication and to illustrate how insights from health literacy can augment recommended methodological approaches and support greater clarity.

## 2. Shape Accessible Information

Some of the difficulties regarding the clarity of our communication in science, medicine, and public health may be attributed to the very ease of communicating. Our society’s ubiquitous computers and phones linked to the internet and printers make publication and dissemination simple and instantaneous. This has fostered the illusion that subject experts can easily be creators and disseminators of scientific and health information, directions, descriptions, questionnaires, and data displays. Scientific and health writing, fact-based and easily verified, can appear almost intuitive to those educated in the field. This faulty assumption of ease may lead to a lack of scientific rigor in the approach to messages and materials prepared for the public.

However, the fields of public health, health education, communication, and marketing have long included a range of research protocols to assure that programs, messages, and materials are designed to meet the needs of the intended audience. Each of these fields has articulated similar processes and methods. The following sections address how health literacy studies can augment critical components of a rigorous formative research approach and thereby further improve information for the public.

## 3. Expand Demographic Data

Information about the intended audience has long been considered the foundation stone in health education, communication, and marketing. Findings from literacy surveys and health literacy studies should now augment the list of commonly gathered demographic information. Literacy skills must be added to the profile of the intended audience. Faulty assumptions about literacy skills of adults must be corrected. We now know from almost three decades of research that a significant proportion of adults in most industrialized nations have difficulty using commonly available materials to accomplish mundane tasks.

Assessments of the literacy and numeracy skills of adults in industrialized nations were undertaken in the early 1990s and have been reassessed regularly since. The original purpose of the surveys was to ascertain the public’s readiness to participate in the complex social and economic environments of the 21st century [7]. Over the years, the survey content has expanded to include problem solving as well as technological skills. The number of participating nations has increased as well, from the initial 22 member nations of the Organization for Economic Cooperation and Development (OECD) to over 40.

The publication of results from the initial surveys conducted in the 1990s caused shock and dismay in the education and economic sectors and served to challenge the faulty assumption that universal schooling yields highly literate societies. Findings indicated that large percentages of adults had difficulty using commonly available materials with accuracy and consistency—and still do. The 2019 report Skills Matter, notes that significant numbers of adults with low literacy, numeracy, as well as technological and problem solving skills are still to be found across all participating industrialized nations. On average, adults’ skills in many OECD nations, including the U.S., are sub-par. Findings offer strong indications that the literacy skills of adults are not adequate to meet the expectations and demands of work and civic engagement [8].

These findings add literacy as a new variable to the mix of key factors that offer an understanding of the audience. Those of us who provide the ‘texts’ for the public, whether written, spoken, posted on line, illustrated in diagrams and charts, or sent via mobile phone, have a responsibility to become familiar with these findings to better understand and communicate with the public. Research from the education field should inform and help shape our efforts to provide information.

## 4. Apply Research Findings

Despite the well documented evidence of limited or low literacy and numeracy skills among adults in the U.S. [9], insufficient action has been taken by those of us preparing and disseminating scientific-, environmental-, and health-related information. For example, over a decade after the first adult literacy analyses were made available in the U.S. in 1993, more than 2000 peer reviewed health literacy studies indicated that health materials are generally written at levels of complexity far beyond the reading skills of average high-school graduates [10]. Then, and subsequently, materials under study included critical medical texts, statements of rights and responsibilities, research tools such as questionnaires, and report back information for study participants.

Critical public health alerts and calls for preparation or action in times of crisis or in the aftermath of disaster have been shown to be similarly problematic. For example, in the U.S. shortly after a major terrorist attack, information regarding an anthrax threat was mailed to every residence in the country to promote efficacious action. However, the very brief material contained formal and vague vocabulary and generated public confusion [11]. Overall, published studies indicate that a majority of health materials under study are unnecessarily complex and ill-suited for the general public [12].

Accessible information is critical for those holding responsibility for communicating directly with the public as well [13]. In the aftermath of the 2011 Fukushima disaster involving an earthquake, a tsunami, and a nuclear plant failure, public health, medical, and nursing professionals were often tasked with ‘translating’ scientific information for community residents such as parents who wanted to know if their children could safely play outside. Many professionals were unable to do so. Even though Japan has the highest mean literacy proficiency scores among the nation members of the OECD, nurses, teachers, and others faced difficulty answering questions about radiation [14]. Adequate ‘translations’ from scientific language into everyday language was missing at each step. Scientists presented findings to policymakers. Policymakers sent that same information to practitioners. Practitioners who interacted with the public provided that same information in the same language and, in so doing, lost the trust of community members. Translators were sorely needed at each step. Clear information, crafted in plain language, can improve the readiness of all—including professionals—to understand, discuss, and present environmental health findings and concepts to one another.

## 5. Follow Rigorous Protocols

The literacy skills of the public and the assessed quality of scientific and health information provided to the public are both well documented; so too is the mismatch that renders much of that valuable information inaccessible. Researchers and practitioners in the fields of health education, health promotion, health communication, as well as marketing have contributed to a substantial body of literature offering text books, guidelines, and how-to-manuals articulating formative research methods that can serve to alleviate part of this mismatch [13,15]. Health literacy insights add to these resources.

As noted above, information about literacy and numeracy skills of the intended audience offer new demographic variables, but this understanding also offers insight for the development of data collection instruments as well as for pilot testing protocols and follow-up revisions. Questionnaires, discussion groups, intercept interviews, and focus groups must be designed to account for literacy. For example, insights from literacy studies tell us that the pilot testers should always include people with average or limited literacy skills. This calls for additional protocols so that participants are never made to feel uncomfortable [15]. Consequently, suggested strategies include having facilitators always read any texts aloud, no matter the composition of the group. The facilitators can ask participants to follow along and use highlighters to identify words, phrases, or sections that a neighbor or friend would have trouble understanding. In addition, pilot test participants can be asked to identify the meanings they attribute to topical vocabulary. This can mediate issues related to different meanings or interpretations for the same concepts.

Pilot testing must engage members of the intended audience—not just to monitor their reactions, but to involve them as language and cultural informants and as co-developers. These processes do not always need to be extensive. In times of chaos, disaster, or crisis, when information is sorely and quickly needed, pilot testing can be done with small groups, often locally recruited. In addition, a trained staff of ‘first responders’ can be called to action to access and revise materials as needed [11].

At the same time, not all of us have the responsibility for developing messages or materials. Hospitals and health centers, for example, rarely develop their own patient education materials, directives, and forms. These are most often provided by outside vendors. Consequently, some institutions are considering inserting pilot test requirements and reports into contracts with vendors. Many health institutions have been introducing their in-house staff to assessment tools to enable them to make informed choices amongst available resources or to be able to modify materials at hand [16]. Several of the commonly used assessment tools in health literacy work are described below.

## 6. Make Use of Assessment Tools

Health literacy studies and programs have called attention to a variety of freely available assessment tools that offer measures of the suitability of a material as well as insights and guidelines for writing and designing new materials. As tools, they offer measures of the viability of a match between the materials and the intended audience. Attention is brought to a variety of elements that hinder or ease reading, listening, comprehension, and use.

The most frequently used tools are the readability assessments which offer superficial, but useful examinations of word length in the English language. Multisyllabic words in English often contain silent letters that confuse readers. These tools draw attention to the use of words like *utilize* when *use* could do. The SMOG tool, also known as the Simple Measure of Gobbledygook [17], is the only readability tool that focuses on both word and sentence length. Long sentences in any language often contain clauses and asides that distract readers away from the main idea. Readability assessments offer a score that indicates reading grade level (RGL). This now commonly used scoring schema was initially used to indicate the match between the vocabulary and complexity of sentences found in school textbooks with the expectations for students’ abilities in a given grade. The RGL was often printed on books for children. It is currently used as a measure of difficulty. The RGL score enables developers to test if their materials score at 8 or below, considered a reasonable match with the reading skills of an average high school graduate. If not, a fix draws attention to modifying vocabulary in use and shortening long sentences. None of the readability tools capture jargon, technical words, or complex concepts (such as risk) that are not multisyllabic. In addition, the tools do not attend to other aspects of a text that can aid or hinder reading and comprehension.

Several tools are available for a more in-depth assessment. The Suitability Assessment of Materials (SAM) was the first tool to move beyond readability and pay attention to myriad key components of a text that ease or restrict reading. This includes attention to and scoring of literacy demand (RGL), but also examines organization, relevance of content, graphic components, layout and typography, the presence of learning motivations and prompts, and the cultural appropriateness of the materials [15]. Each element is fully discussed and detailed criteria for scoring is presented.

Inspired by the SAM, scholars at the Center for Disease Control designed the Clear Communication Index (CCI) to help writers assess some of the same areas of concern, with additions most pertinent to public health such as a score for state of the science and for the explanation of risk [18]. The CCI offers a vehicle for measuring the clarity of the main message and insuring that a call to action is included and prominent. The CCI also includes measures of design and organization, language clarity and appropriateness for the intended audience, the use of numbers, as well as ratings for the inclusion and clarity of behavioral recommendations. The online posting includes an orientation, training program for use, and a detailed explanation of scoring.

The Agency for Healthcare Research and Quality’s Patient Education Materials Assessment Tool (PEMAT) was somewhat similarly designed to shorten the SAM process, but go beyond a RGL. The measures include attention to word choice, style and organization, use of numbers, layout and design, and clarity of content. An additional component focuses on the use of visual aids and videos [19]. The online posting includes an orientation and in-depth training program to assure understanding of the elements to be examined and scoring criteria.

The SMOG, SAM, CCL, and PEMAT are all focused on prose materials—information structured in sentence and paragraph format. However, none of these tools focus on data displays which are of frequent use and of key importance in science, medicine, and public health. The PMOSE/IKIRSCH tool [20], was developed for the education field and focuses on materials referred to as ‘documents’ (materials providing words and/or numbers in isolation) such as lists, charts, graphs and other data displays. The tool offers a measure of the complexity of these materials with a focus on type, density, and dependability (a negative factor indicating a need to find information outside the display). The overall score, in the form of a grade level, offers an indication of the complexity and suitability of the materials.

Lacking thus far are more refined instruments for judging the quality of the presentation of data and use of numbers commonly found in reports of medical test findings or in environmental study results. Several health literacy studies and analyses offer insights for the best use of numbers and display times that ease the burden for users. For example, Apter and colleagues urge researchers, practitioners, and public speakers to refrain from switching amongst and between whole numbers, fractions, numbers with decimal points, and percentages. They not only point out the need for consistency in how numbers are presented, but note that we should do the math for people and not expect them to do calculations or transformations [21]. Anker and Zigmund-Fisher offer insights into how displays are best constructed (such as with the use of icons) and suggest that numbers always be offered in context with clear indications of when action is needed [22,23].

Currently, we also lack instruments for measuring the suitability of forms, questionnaires, and web postings. However, multiple guidelines such as those related to assessing web sites are readily available on line at U.S. government websites and can be transformed into checklists for assessment purposes [24]. Once new assessment instruments do emerge, we must add them to the toolbox.

Overall, the currently available assessment tools offer measures indicating weak points in a text, posting, or presentation. They provide valuable insight to inform design, offer a comparative measure, and give valuable insight for rewriting and restructuring. These tools could, and perhaps should, be appropriately modified to address the specific needs of those developing forms, reports, data displays, and educational materials specifically for environmental health issues, measures, and vocabulary.

Finally, formative research involves new rounds of revisions post-assessment. The development of health information, taken seriously, is a rigorous process. These steps—generally applied to the development of medicines, procedures, products, or devices are universally accepted as necessary components of the scientific method.

## 7. Conclusions

Environmental studies, always a key component of public health, has now emerged as a major discipline for addressing our most pressing global concerns. As is noted above, environmental health communication and most science communication can be challenging. For example, in order to access information about clean water, community residents often have to navigate scientific terms, measures presented in unusual format (parts per), complex charts, as well as the sometimes arcane legal vocabulary found in legislative regulations [25]. A rigorous approach to information design, message development, testing, and revision could help mitigate problems for the lay public and make needed information more accessible.

In this contentious era of science skepticism, more readily available plain language translations of information related to the environment, climate crisis, global health threats, preparation for and after-effects of natural disasters—could make a difference. It is time that we, along with policy makers, grant funders, institutional contractors, and peer reviewers insist on rigorous formative research and ‘translation’ practice for the dissemination of information for the public. Publishing rules and regulations, funding contracts, and scholarly expectations can establish needed normative change to make readily available information more accessible.

The development of health information, taken seriously, ought to be an accepted rigorous process. Unfortunately, protocols universally accepted as necessary components of the scientific approach are not always followed for the development of health information. Arguments against such practices cite costs and time issues. However, neither cost-related nor time-constraint arguments are acceptable explanations for bypassing rigor or tolerating shortcuts in the scientific arena. Such arguments are not to be brooked for the important effort to share information, build knowledge, and prepare the public for action.

Public health efforts, in general, and environmental health goals, specifically, rely on an informed citizenry. If scientific findings, evidence of health consequences, risk analyses, local and national policy options, and/or emerging issues are available but not accessible, then action as well as knowledge is hampered. Findings from the fields of education and literacy indicate that ‘practice as usual’ will leave large segments of the population behind. We must be aware of our audience, take the word seriously, and shape our information with rigor. We need to be scientific in our approach.
